# Competitive virus and host RNAs: the interplay of a hidden virus and host interaction

**DOI:** 10.1007/s13238-014-0039-y

**Published:** 2014-04-12

**Authors:** Changfei Li, Jun Hu, Junli Hao, Bao Zhao, Bo Wu, Lu Sun, Shanxin Peng, George F. Gao, Songdong Meng

**Affiliations:** CAS Key Laboratory of Pathogenic Microbiology and Immunology, Institute of Microbiology, Chinese Academy of Sciences (CAS), Beijing, 100101 China

**Keywords:** cvhRNAs, virus RNAs, host mRNAs, miRNA response element, ceRNAs

## Abstract

During virus infection, viral RNAs and mRNAs function as blueprints for viral protein synthesis and possibly as pathogen-associated molecular patterns (PAMPs) in innate immunity. Here, considering recent research progress in microRNAs (miRNAs) and competitive endogenous RNAs (ceRNAs), we speculate that viral RNAs act as sponges and can sequester endogenous miRNAs within infected cells, thus cross-regulating the stability and translational efficiency of host mRNAs with shared miRNA response elements. This cross-talk and these reciprocal interactions between viral RNAs and host mRNAs are termed “competitive viral and host RNAs” (cvhRNAs). We further provide recent experimental evidence for the existence of cvhRNAs networks in hepatitis B virus (HBV), as well as Herpesvirus saimiri (HVS), lytic murine cytomegalovirus (MCMV) and human cytomegalovirus (HCMV) infections. In addition, the cvhRNA hypothesis also predicts possible cross-regulation between host and other viruses, such as hepatitis C virus (HCV), HIV, influenza virus, human papillomaviruses (HPV). Since the interaction between miRNAs and viral RNAs also inevitably leads to repression of viral RNA function, we speculate that virus may evolve either to employ cvhRNA networks or to avoid miRNA targeting for optimal fitness within the host. CvhRNA networks may therefore play a fundamental role in the regulation of viral replication, infection establishment, and viral pathogenesis.

## Introduction

Currently, viral RNAs are regarded as either templates for translating viral proteins that exert function or as viral pathogen-associated molecular patterns (PAMPs) for detection by host pattern-recognition receptors (PRRs) in innate antiviral immunity (Goubau et al., 2013). However, several recent studies provide intriguing evidence that viral RNAs and host mRNAs with common microRNA (miRNA) binding sequences reciprocally affect each other’s levels and activities by directly competing with the targeting miRNAs. In this review, we propose a “competitive viral and host RNAs” (cvhRNAs) hypothesis, including recent studies on miRNAs and noncoding RNAs (ncRNAs) expressed by both the host and virus, as well as the logic of this new and potentially predictable virus-host interaction.

## Mirnas and competitive endogenous rnas (CERNAS)

### MiRNAs

MiRNAs are a class of small noncoding double-stranded RNA molecules of approximately 22 nt in length, which regulate (usually inhibit) target gene expression at the post-transcriptional level. Full-length miRNAs (pri-miRNAs) are transcripted by RNA polymerase II, which are processed by Drosha within the nuclear compartment to produce pre-miRNAs of about 65 nucleotides in length. The pre-miRNA is then transported into the cytoplasm and further cleaved by the RNAse III-like endonuclease Dicer to produce their mature form miRNAs (Newman and Hammond, 2010). Upon loading into the RNA-induced silencing complex (RISC), which contains a member of the double-stranded RNA binding protein Argonaute family (Ago), the guide strand of the miRNA duplex recognizes and binds to conserved complementary target sites in target mRNAs (often in the 3′-UTR) through canonical base-pairing between the seed region of approximately 6- to 8- oligonucleotides. MiRNAs regulate target gene expression either by inducing their deadenylation and degradation or by leading to translational inhibition (Miyoshi et al., 2009; Hu et al., 2012). Thus far, >2500 human miRNAs (hsa-mir) and 1900 mouse miRNAs (mmu-mir) have been identified and described at the miRBase website (http://www.mirbase.org, released in Nov. 2013).

### CeRNAs

Aside from small regulatory ncRNAs, such as miRNAs, the transcription machinery in mammalian cells also produces long noncoding RNAs (lncRNAs) which are typically >200–300 nt in length. Although currently the function of most of the annotated lncRNAs remains unknown, emerging studies show that certain lncRNAs are involved in various physiological processes, including cell-cycle regulation, apoptosis, and the establishment of cell identity (Ulitsky and Bartel, 2013). Functional lncRNAs may act through several mechanisms, including co-transcriptional regulation, *cis*- or *trans*-regulation of gene expression by bridging proteins and chromatin, titration of RNA-binding factors, and pairing with other RNAs to trigger post-transcriptional regulation (Ulitsky and Bartel, 2013; Augui et al., 2011). Poliseno et al. (2010) further uncovered a unique interaction of the PTEN tumor suppressor gene and its pseudogene (PTENP1), both of which are subject to the same miRNA-mediated post-transcriptional regulation (Poliseno et al., 2010). Pseudogenes harbor premature stop codons, deletions/insertions, and mutations that abrogate their translation into functional proteins. Interestingly, the PTENP1 3′UTR functions as a decoy for PTEN-targeting microRNAs due to its ability to compete for miRNA binding and thus exerts a tumor-suppressive role by modulating the de-repression of cellular levels of PTEN. A subsequent study shows that a putative PTEN competitive endogenous RNA (ceRNA), the ZEB2 transcript, which contains common miRNA recognition elements with PTEN, modulates PTEN protein levels in a protein coding-independent manner (Karreth et al., 2011). These studies indicate a new means of regulatory interaction between mRNAs (including both protein-coding and non-coding mRNAs) that have common microRNA recognition elements.

Further, more recent studies provide compelling evidence that the crosstalk between ceRNAs through competition for their shared miRNAs is involved in signaling pathways and networks in human diseases, such as autoimmune diseases, diabetes and cancer (Ling et al., 2013a, b; Fang et al., 2013; Noorbakhsh et al., 2013; Karreth and Pandolfi, 2013). Together, these newly defined mRNA-mRNA interactions have integral roles in the regulation of gene expression in various physiological and pathological processes and deepen our understanding of a novel aspect of RNA function.

## The cvhrnas hypothesis

**Figure 1:**
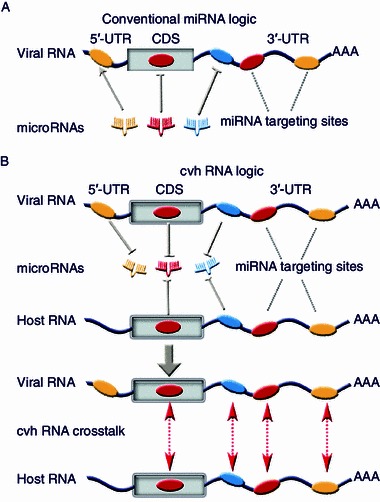
**Inference of cvhRNA cross-regulation through miRNA response elements**. (A) MiRNAs guide the recognition of target mRNAs through imperfect matches with their targeting sites and thus regulate the expression of target mRNAs at the post-transcriptional level. Each miRNA has multiple (up to tens) mRNA targets. Conversely, each mRNA may harbor multiple miRNA targeting sites (Ala et al., 2013). (B) Viral RNAs and mRNAs harboring miRNA response elements exert their suppression of miRNA as sponges. Host mRNAs may act in a similar way. Thus, viral RNAs and host mRNAs could competitively sequester the same miRNA pool within infected cells. Viral RNAs as miRNA sponges can de-repress the miRNA-mediated inhibition of host mRNAs. Therefore, a cross-regulation may be formed between viral RNAs and host mRNAs that share common miRNA response elements. Stimulation (↑) or inhibition (┬) is determined following how miRNAs or RNAs impact the activities of RNAs or miRNAs

Somewhat differently from the mechanism mentioned above and unlike cellular endogenous RNAs, viral RNAs and mRNAs are exogenous in virus-infected cells. In addition, viral RNAs are mostly studied as passive targets for host miRNAs (Fig. 1A). Indeed, a large number of impressive experiments have been performed, primarily on binding site validation and functional analyses, which support the notion that the interaction between miRNAs and viral RNAs as their targets is essential to the regulation of viral RNA stability, expression, and translation, as well as viral replication and infection (Jopling et al., 2005; Shimakami et al., 2012; Song et al., 2010; Klase et al., 2012). Hosts employ their miRNAs in defense against viral infection by targeting viral products or inhibiting viral replication. Whereas viruses also encode miRNAs that target specific host genes and pathways to prolong the longevity of infected cells or evade the immune clearance, which may be beneficial for viral infectivity and/or proliferation (Carl et al., 2013; Kincaid and Sullivan, 2012).

Nevertheless, viral RNAs harbor common miRNA-binding sites with host RNAs (Jopling et al., 2005; Shimakami et al., 2012; Song et al., 2010; Klase et al., 2012), indicating that viral RNAs and mRNAs potentially act as bona fide miRNA competitors with host RNAs for the same miRNA pool in infected cells. In addition, a large number of virus-encoded miRNAs and lncRNAs have been uncovered, which play a key role in persistent infections, and importantly, a significant portion of viral miRNAs mimic host miRNAs with similar target sites (Kincaid and Sullivan, 2012). Therefore, we hypothesize that viral RNAs act as sponges that can sequester endogenous miRNAs within infected cells and thus impact the stability and translational efficiency of host mRNAs with shared miRNA response elements (Fig. 1B). We term the reciprocal regulation between virus and host transcripts acting in this manner cvhRNAs.

The basis for cvhRNA crosstalk is dependent on base pairing complementarity and miRNA binding/recognition, and the ability of viral RNAs (or host mRNAs) to sequester and degrade miRNAs and the extent of de-repression in host mRNAs (or viral RNAs) by miRNA down-regulation. Down-regulation of miRNA by interacting viral or host mRNAs could be either via sequestration/sponge or destabilization/degradation, or both (Torres et al., 2011), although the exact mechanisms await further investigation. Thus, the relative abundance of virus vs. host RNAs, levels of common miRNAs, and the number of miRNA response elements may all contribute to cvhRNA interactions, according to a mathematical mass-action model for ceRNA networks (Ala et al., 2013). In addition, the intracellular localization of virus/host mRNAs and miRNAs, the extent of base pairing complementarity of the competitive RNAs, as well as the time-course of viral RNA expression may also impact ceRNA networks. Given that viruses usually generate highly redundant transcripts upon infection and that multiple highly abundant miRNAs interact with viral RNAs or mRNAs (Shimakami et al., 2012; Kincaid and Sullivan, 2012), we expect that reciprocal interactions between viral RNA-miRNA-host mRNA may orchestrate a permissive molecular environment that enables the generation of cvhRNA networks under virus infection.

## The experimental evidence for cvhrna networks

### The existence of cvhRNA cross-talk in viral infections

An early indication that virus-encoded RNAs are involved in regulating host miRNA abundance came from Cazalla et al. (2010), who demonstrated that *Herpesvirus saimiri* (HVS)-expressed noncoding RNAs have certain miRNA binding elements and can interact with host miRNAs in virally transformed T cells. They found that viral ncRNAs down-regulate mature miR-27 in a binding-dependent manner. Transient knockdown of one of the ncRNAs, HSUR 1, resulted in a decreased level of the miR-27 target protein, FOXO1 (Cazalla et al., 2010). Subsequently, several impressive experiments have been performed on the role of coding RNAs and ncRNAs on the degradation and decay of host miRNAs. Lytic murine cytomegalovirus (MCMV) infection leads to rapid degradation of cellular miR-27a and miR-27b via expression of a spliced and highly abundant protein-coding transcript, m169, which facilitates efficient virus replication (Marcinowski et al., 2012). Interestingly, MCMV infection up-regulates expression of genes in mitogen-activated protein kinases (MAPK) pathway, specifically p38, which enhances viral replication (Tang-Feldman et al., 2013). Up-regulation of p38 may attribute to decreased miR-27a as p38 is a potential target of miR-27a. Similarly, a viral intergenic ncRNA element of human cytomegalovirus (HCMV) mediates the selective down-regulation of mature miR-17 and miR-20a through sequence-specific interactions. Viral RNA-induced miR-17 decay is essential for rapid viral production, indicating a potential role for the interaction between the viral ncRNA and cellular miRNAs during viral pathogenesis (Lee et al., 2013a). Indeed, IL-8 as a target of miR-17/20a (Yu et al., 2010), significantly stimulates HCMV replication through cytokine-stimulated transcription of viral late genes and activation of early events in viral replication (Szabó et al., 1999; Redman et al., 2002).

Hepatitis B virus (HBV), a major hepatotropic DNA virus, chronically infects ~350 million people worldwide. Cellular miRNAs have a striking role in mediating HBV replication and infection (Hu et al., 2012; Zhang et al.,^2013^). MiR-122 is the most abundant liver-specific miRNA, which reaches approximately 70% of the total miRNA population in the adult liver. We observed a significant decrease of miR-122 levels under HBV infection. We further demonstrated that a miR-122 binding element exists in the 3′-UTR of all four HBV mRNAs (i.e., the pre-C/C or pre-genomic RNA (pgRNA), pre-S, S, and X mRNAs), and these viral RNAs act as sponges to bind and sequester endogenous miR-122, which led to decreased miR-122. Furthermore, expression of the HBV 3′-UTR-containing luciferase reporter was sufficient to up-regulate expression of two cellular genes, cyclin G1 and pituitary tumor-transforming gene 1 (PTTG1) binding factor (PBF), whose 3′-UTRs contain a common miR-122 response element as HBV RNAs (Li et al., 2013; Wang et al., 2012). A follow-up study revealed that cyclin G1 significantly enhances HBV expression and replication by suppressing p53-mediated inhibition of HBV enhancers, indicating that the cross-regulation of HBV RNAs and cyclin G1 expression possibly contribute to viral persistence. Further, up-regulation of PBF may contribute to HBV infection-related hepatocellular carcinoma (HCC) because PBF interacts with PTTG1 and increases its transcriptional activity at multiple oncogenes (Li et al., 2013), as well as promotes cell proliferation, invasion, and HCC tumor growth in mice. Under HBV infection, the HBV RNA copy number per cell reaches up to 105 (Li et al., 2013; Wang et al., 2012), and miR-122 is expressed in hepatocytes at 50,000 copies per cell (Hu et al., 2012; Filipowicz and Grosshans, 2011). Conceivably, a positive cross-regulation between HBV RNAs and cyclin G1 or PBF occurs in the presence of the large amounts of viral RNAs which efficiently sequester miR-122, resulting in intermediate miR-122 levels in hepatocytes.

Interestingly, we also identified an IFN-stimulated gene (ISG), NT5C3, which efficiently sequesters miR-122 with its mRNA 3′-UTR through binding sequence-specific interactions (Hao et al., 2013). Conceivably, the possible HBV RNAs-NT5C3 mRNA crosstalk may be a counter mechanism that HBV evolved to antagonize the antiviral activity of IFN.

In addition to the interaction between HBV RNAs and host mRNAs that share a common miR-122 response element, two other groups more recently reported another example of cvhRNA crosstalk in HBV infection (Liu et al., 2013; Wang et al., 2013b). They found that the HBx transcript and HBV mRNAs directly trigger the down-regulation of miR-15a/16 via the miRNA targeting sequences in the viral RNA, and as a consequence, the target of miR-15a/16 (Bcl-2) is up-regulated. Up-regulated Bcl-2 then inhibits the downstream cascade of apoptosis in hepatoma cells. These studies identify a novel HBx transcript and the HBV mRNAs-miR-15a/16-Bcl-2 regulatory pathway that is involved in apoptosis inhibition, which may help facilitate HBV infection and HCC development.

**Figure 2:**
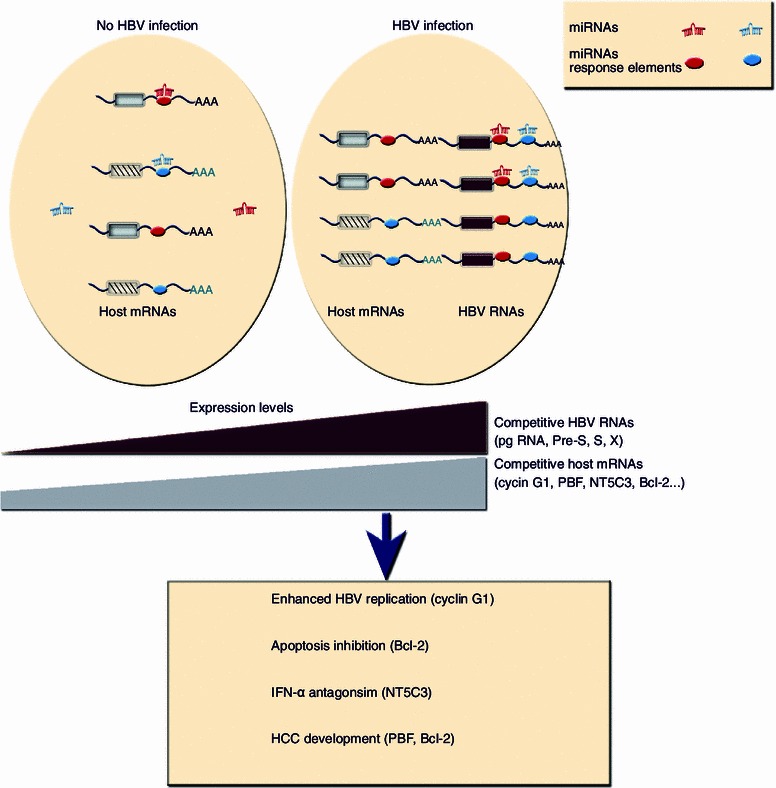
**Schematic figure of how cvhRNA networks may mediate viral replication, infection, and the development of HCC during HBV infection**. In hepatocytes, miR-122 effectively suppresses the expression of its target genes (cyclin G1, PBF, and NT5C3) at the post-transcriptional level by binding to the miRNA response element within the 3′-UTR of target mRNAs. During HBV infection, high levels of viral RNAs harboring a miR-122 response element in their 3′-UTR competitively sponge and efficiently sequester cellular miR-122, thus blocking the binding of miR-122 to its host target mRNAs. In this manner, viral RNAs de-repress and rescue the expression of host target mRNAs. Similarly, HBV RNAs positively cross-regulate Bcl-2 expression through shared miR-15a/16 response elements. The dose effect between competitive HBV RNAs and host mRNAs is shown. In the networks consisting of four viral RNAs (pgRNA, pre-S, S, and X mRNAs) and four host mRNAs (cyclin G1, PBF, NT5C3, and Bcl-2), elevated expression of de-repressed host genes contributes to enhanced HBV replication, persistent viral infection, and HCC development

Together, the above studies provide experimental evidence for the existence of cvhRNA networks during HBV infection (Fig. 2). CvhRNAs, including HBV RNAs (e.g., pgRNA, pre-S, S, and X mRNAs) and host mRNAs (e.g., cyclin G1, PBF, NT5C3, and Bcl-2), harbor shared response elements for certain abundant cellular miRNAs (e.g., miR-122 and miR-15a/miR-16). When viral RNAs are highly produced during HBV infection, the repression of host target genes conferred by these common miRNAs can be diluted as viral RNAs down-regulate common miRNAs. Up-regulation of host target genes would contribute to enhanced HBV expression and replication, persistent viral infection, and HCC development.

HCV (hepatitis C virus), another main hepatotropic positive-sense RNA virus, causes chronic infection in about 180 million people worldwide. Extensive studies have demonstrated that miR-122 acts in an unusual manner to stimulate the replication and expression of HCV by binding to two closely spaced target sites in the 5′-UTR of the HCV genome (Jopling et al., 2005; Shimakami et al., 2012; Pang et al., 2012). In this context, interaction of miR-122 and viral RNA stabilizes viral RNA and decreases its decay, rather than induces its degradation as most miRNAs do. Notably, Shan et al. (2007) showed that inhibition of miR-122 up-regulates Heme oxygenase-1 (HO-1) which significantly inhibits HCV replication, suggesting that miR-122 promotes HCV replication partly via down-regulation of HO-1 (Shan et al., 2007). Considering that HCV RNA also interacts with miR-122, it is possible that the positive regulatory pathway of HCV RNA-miR-122-HO-1 may contribute to enhanced HCV replication.

In HBV and HCV infections, robust viral specific T cell responses are observed during resolution of acute and self-limited HBV infections with viral clearance, whereas chronic infection with viral persistence is characterized by only weak and impaired T-cell response. T cells are therefore believed to play a critical role in the control of HBV and HCV replication and infection (Manigold and Racanelli, 2007; Wang et al., 2013a). Conceivably, cvhRNA networks may play an important role in regulation of host immune responses in the liver microenvironment that induce immune tolerance towards viruses. Indeed, the above preliminary studies have shown that the cross-talk and reciprocal interactions between HBV/HCV RNAs and host mRNAs contribute to enhanced and persistent viral expression and replication which inevitably drive T cell tolerance and exhaustion by long-term intensive viral antigenic stimulation. Moreover, it is possible that cvhRNA networks in HBV or HCV infection may directly or indirectly affect expressions of co-inhibitory molecule programmed death (PD)-1 on T cells and its ligand PD-L1 on dendritic cells (DCs), the cytotoxic T lymphocyte antigen-4 (CTLA-4) and proapoptotic protein Bcl2-interacting mediator (Bim) on T cells, or activities of Tregs in the liver microenvironment. Further understanding the regulation of cvhRNA networks in influencing the outcome of viral infections (e.g. viral clearance or persistent infection) will allow the development of novel strategies for therapeutic regimen by targeted reversal of tolerising mechanisms.

### Prediction of potential cross-regulation between host and virus by cvhRNA hypothesis

In principle, any viral RNAs sharing common miRNA response elements with host mRNAs could potentially act as cvhRNAs and form regulatory networks across the transcriptome. Besides HSV, MCMV and HCMV, we speculate that other examples of cvhRNA may also exist in viral infections (Table. 1). For instance, HIV-1 may probably up-regulate Notch-3 through cvhRNA crosstalk, as both HIV-1 gag-pol and Notch-3 mRNAs share a common miR-150 response sequence (Huang et al., 2007; Ghisi et al., 2011), and HIV-1 infection down-regulates miR-150 (Swaminathan et al., 2009). Notch activation in turn is involved in HIV-associated nephropathy (Sharma et al., 2010). The cvhRNA hypothesis also predicts another possible reciprocal interaction between HIV nef and host PTEN mRNAs that have a shared miR-29a response sequence. HIV infection may induce apoptosis of CD4^+^ T lymphocytes through upregulating PTEN (Kong et al., 2011; Sun et al., 2012; Dabrowska et al., 2008). Similarly, influenza virus WSN infection induces up-regulation of MMP9 which mRNA shares a common miR-491-5p binding site with viral PB1 mRNA (Song et al., 2010; Lee et al., 2013b; Yuan et al., 2013), indicating the potential cross-regulation between viral mRNA and host mRNA. Furthermore, the notion of cvhRNAs was recently linked to human papillomaviruses (HPVs) (Gunasekharan and Laimins, 2013). HPV-31, whose E1 and E2 open reading frames contain miR-145 binding sequences, could lead to a dramatic decrease miR-145 level and increased the expression of a miR-145 target, the cellular transcription factor KLF-4. HPV-regulated KLF-4 may contribute to the control of viral life cycle during differentiation.

**Table 1:** Prediction of potential cross-regulation between host and virus by cvhRNA hypothesis

Viruses	Competitive virus RNAs	miRNAs	Competitive host RNAs	Effects of cvhRNAs on viral infection and pathogenesis	References
HIV-1	gag-pol	miR-150	NOTCH3	HIV-associated nephropathy	Huang et al., 2007; Ghisi et al., 2011; Swaminathan et al., 2009; Sharma et al., 2010
	Nef	miR-29a	PTEN	Apoptosis in infected CD4^+^ T Lymphocytes	Kong et al., 2011; Sun et al., 2012; Dabrowska et al., 2008
IAV	PB1	miR-491	MMP-9	Severe lung pathology	Song et al., 2010; Lee et al., 2013b; Yuan et al., 2013
HPV	E1,E2	miR-145	KLF-4	Control of viral life cycle	Gunasekharan and Laimins, 2013
MCMV	m169	miR-27a	p38	Viral replication and atherogenesis	Marcinowski et al., 2012; Tang-Feldman et al., 2013
HCMV	UL144-145 RNA	miR-17/20a	IL-8	Viral replication	Lee et al., 2013a; Yu et al., 2010; Szabó et al. 1999; Redman et al., 2002

As cvhRNA networks primarily depend on viral mRNA abundance, different mechanisms responsible for mRNA production among different virus families above are listed in Table 2. Among these virus types, ssRNA, dsRNA or dsDNA genomes serve as the template for mRNA transcription, some of which even contain a reverse transcription stage in their replication cycle. This indicates that cvhRNA networks may exist universally among viruses with different types of genomes (e.g. ssRNA, dsRNA and dsDNA).

**Table 2:** Different mechanisms for viral mRNA production

Viruses	Genome	mRNA production
IAV	(−)ssRNA	(−)vRNA → mRNA (−)vRNA → (+)cRNA → (−)RNA → mRNA
HCV	(+)ssRNA	(+)vRNA → mRNA (+)vRNA → (−)RNA → (+)RNA → mRNA
HIV-1	dsRNA	dsRNA $\mathop \to \limits^{\text{RT}}$ dsDNA → mRNA
HBV	dsDNA	DNA → mRNA DNA → pgRNA $\mathop \to \limits^{\text{RT}}$ cccDNA → mRNA
HVS	dsDNA	DNA → mRNA
HPV	dsDNA	DNA → mRNA
HCMV	dsDNA	DNA → mRNA
MCMV	dsDNA	DNA → mRNA

## Conclusions and outlook

In conclusion, we speculate that cross-talk between viral RNAs and host mRNAs via common miRNA response elements forms complex networks during virus infection, which may affect viral replication, persistence, and pathogenesis. This expands the knowledge of how viral RNAs and mRNAs function in multiple ways, not merely as blueprints for viral protein synthesis or as PAMPs in innate immunity. Figs. 1 and 2 only show the direct interactions between viral RNAs and host mRNAs. In general, one miRNA has several or even multiple RNA/mRNA targets, and conversely, one target RNA or mRNA likely harbors several miRNA response elements. Thus, indirect interactions between viral RNAs and host mRNAs may also play an important role in cvhRNA networks, which may be further intimately intertwined by cellular transcription factor networks (Ala et al., 2013). In addition, variations in miRNA response elements (e.g. the seed region of pairing) of viral RNA which inevitably affect base pair complementarity, could thereby influence the interactions between viral and host RNAs. To establish infection, viruses likely evolved to accumulate mutation to increase the number of shared miRNA response elements with supportive host factors and decrease the number shared with inhibitory host factors; this hypothesis deserves further investigation. Global mRNA and miRNA sequencing, Gene Ontology analysis of transcriptomic databases and establishment of proper mathematical models are needed to provide further dissection of the impact of cvhRNA networks on virus and host interaction.

**Figure 3:**
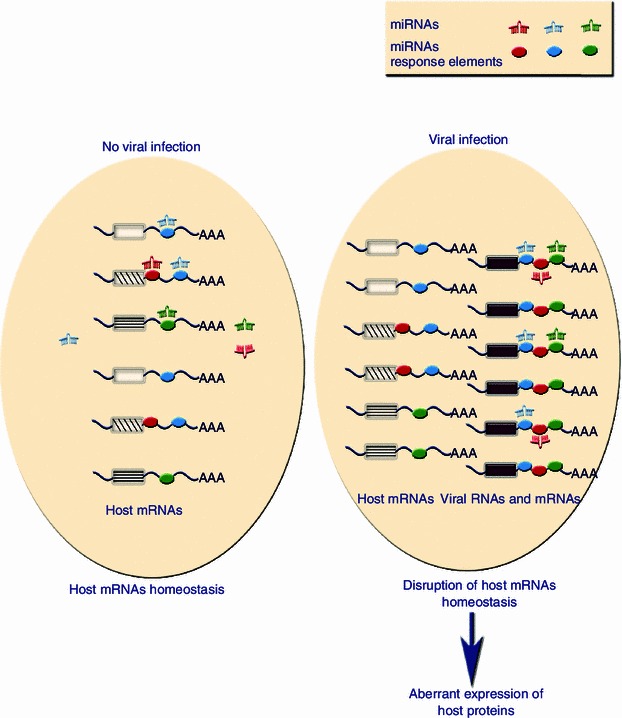
**Potential alteration of host mRNA homeostasis by viral RNAs**. Many mammalian viruses produce high levels of redundant viral RNAs and mRNAs. According to the cvhRNAs hypothesis, under certain conditions for cvhRNA activity in viral infections, these viral RNAs could modify the balance between cellular miRNAs and host mRNA targets in a miRNA response sequence dependent and coding independent manner. Viral RNAs directly result in the depression of cellular transcripts with shared miRNA response elements and the subsequent aberrant expression of host proteins

A fine-tuned balance between miRNAs and host mRNA targets (including coding and noncoding transcripts) likely exists in cells with no viral infection (Ala et al., 2013). Conceivably, viral infection disturbs mRNA homeostasis through cvhRNA networks, as shown in Fig. 3. Information regarding the balance between miRNA pools and host mRNA homeostasis under viral infection is essential to understand the molecular basis for viral persistence and pathogenesis, which may facilitate the development of new antiviral strategies.

An intriguing question arises from the hypothesis of cvhRNA regulatory networks is why virus evolves to facilitate rather than avoid miRNA binding which inevitably leads to repression of viral RNA function. We speculate that virus may employ either strategy (to usurp cvhRNA networks or to avoid miRNA targeting) for the establishment of persistent infection, depending on which strategy plays a major role in favoring viral infectivity and replication. In particular, some viruses produce redundant RNA/mRNAs during infections as seen in HBV infection (Li et al., 2013; Wang et al., 2012), which facilitate to form cvhRNA networks.

As emerging studies reveal that cvhRNAs play essential roles in viral infection and pathogenesis, it will be important to take into consideration the unique features of direct communication between viral RNAs and host mRNAs when studying the interaction between the virus and host in the long run. We hope that this review of recent advances on ceRNA and cvhRNA networks will serve to stimulate more interest and experimental activity and re-define the concept of virus-host interactions as more than protein-mediated regulation.

## Abbreviations

ceRNAs: competitive endogenous RNAs
cvhRNAs: competitive viral and host RNAs
PAMPs: pathogen-associated molecular patterns
ncRNAs: noncoding RNAs
HVS: Herpesvirus saimiri
MCMV: cytomegalovirus
HCMV: human cytomegalovirus
HPVs: human papillomaviruses
